# Respiratory diseases call for special attention from clinical and translational science

**DOI:** 10.1186/2213-0802-1-1

**Published:** 2013-02-22

**Authors:** Chunxue Bai

**Affiliations:** Shanghai Respiratory Research Institute, Respiratory Research Institute, Department of Respiratory Medicine, Zhongshan Hospital, Fudan University Shanghai School of Medicine, Shanghai, China

**Keywords:** Translational, Respiratory, Medicine, Diseases, Publication

## Abstract

Respiratory diseases will become one of the top 3 leading causes of estimated mortality in 2020 and become about one third of total causes of estimated mortality. The journal of *Translational Respiratory Medicine* is a truly international, peer-reviewed journal devoted to the publication of articles on outstanding work with translational potentials between basic research and clinical application to understanding respiratory disease. Translational respiratory medicine will more focus on biomarker identification and validation in pulmonary diseases in combination with clinical informatics, targeted proteomics, bioinformatics, systems medicine, or mathematical science; on different translational strategies of cell-based therapy to clinical application to treat lung diseases; on targeted therapies in combination with personalized medicine; and on distant electronic medicine to monitor a large population of people’s health. *Translational Respiratory Medicine* is an additional but unique opportunity for scientists and clinicians who work on pulmonary diseases to publish their outstanding findings, initiative results, and critical and perceptive opinions in the journal.

The journal of *Translational Respiratory Medicine* is a truly international, peer-reviewed journal devoted to the publication of articles on outstanding work with translational potentials between basic research and clinical application to understanding respiratory disease. The journal provides a forum for exchange of ideas on potential molecular and cellular mechanisms and novel treatment for respiratory diseases. *Translational Respiratory Medicine* aims to play an important, critical, and recognized role in the understanding of human respiratory diseases and improvement of the prognosis of patients.

Respiratory diseases need global attentions and solutions now more than ever, especially from clinical and translational medicine. Respiratory diseases have become the second leading cause of mortality with over 130 major causes according to the WHO Global Burden of Disease 2000 study’s (GBD 2000) estimates of incidence, health state prevalence, severity and duration, and mortality for 17 sub-regions of the world [1]. Four out of the top ten leading causes, accounting for approximately 43% of mortality are respiratory diseases such as lower respiratory infections, chronic obstructive pulmonary diseases (COPD), and lung cancer. COPD, respiratory infection and lung cancer will become the top 3 to 5 leading causes of estimated mortality in 2020 and become about one third of total causes of estimated mortality in the world, according to the World Health Report 2002 in WHO website at http://www.who.int/evidence/bod. The journal of *Translational Respiratory Medicine* is an efficient and effective communication channel for publishing articles from leading research scientists and clinicians at the forefront of translational medicine and bridge basic research and clinical application, with a special focus on respiratory diseases. *Translational Respiratory Medicine* becomes the premier source of information in the field of translational medicine in respiratory diseases and one of the leading platforms to translate basic sciences into clinical use, and clinical imaging into bioinformatics.

There is solid evidence that translational research on respiratory diseases has increased greatly. Most current journals related to pulmonology focus on either molecular biology or clinical practices. This means that the most important link between them has been ignored. Translational medicine in respiratory diseases will bring basic research to clinical application and clinical questions to bench. Thus, there is a great need to have a critical and important channel and platform for such communications. *Translational Respiratory Medicine* will promote and accelerate the exchange of bidirectional information on respiratory diseases between basic and clinical researchers, innovators and clinicians, scientists and politicians, and doctors and patients. *Translational Respiratory Medicine,* as a part of clinical and translational medicine, is intended to optimize new methodologies, foster clinical application of new therapeutic strategies, and ultimately improve the quality of life for patients with respiratory diseases [2]. Lung cancer, together with breast, prostate and gastrointestinal cancers, were listed and encouraged as a program of translational research in the National Institutes of Health guide for Specialized Program of Research Excellence grants for cancer research in 1993 [3], which may be the earliest national program focused on translational research. Translational research on respiratory diseases was one of pioneering examples to understand the process of translational medicine, although this remains a continuous process. For example, local therapy with p53 gene for patients with lung cancer was considered as an important milestone in translational cancer research in 1996 [4]. Translational science was initially pointed out as a two-way process between the bench and clinical application. Translational respiratory medicine opens the opportunity for pulmonary scientists and clinicians to fight a number of challenges to translate molecular understanding into clinical applications for lung diseases.

Biomarkers have been defined as an important factor in diagnosing the early phase of diseases, monitoring the severities of the disease and sensitivities to therapies, or even predicting the prognosis of patients with lung diseases. An increasing number of biomarkers are being discovered and identified from preclinical research, while a few can be used clinically. For example, network biomarkers and dynamic network biomarkers, a new type of biomarkers with protein-protein interactions, can be monitored and evaluated at different stages and time points during the development of diseases [5]. Recent study combined genome-wide association with forced expiratory volume in 1 second and the ratio of forced expiratory volume in 1 second to forced vital capacity and identified 16 new regions showing association with pulmonary function [6]. Those biomarkers were selected from 48,201 individuals of European ancestry with follow up of the top associations in up to an additional 46,411 individuals. However, there is further need to better understand those biomarkers associated with molecular mechanisms involving pulmonary function and validate selected targets preventing lung dysfunction. Biomarker identification and validation in pulmonary diseases like COPD stand in the frontline of translational medicine in combination with clinical informatics, targeted proteomics, bioinformatics, systems medicine, or mathematical science [7, 8]. *Translational Respiratory Medicine* is expected to accelerate and standardize the process from post-validation to authority approval, clinical application to policy and regulation, and individual medicine to public health.

Increasing evidence from preclinical studies demonstrated that local or systemic administration of external mesenchymal or induced pluripotent stem cells could prevent or treat lung diseases or improve the severity of diseases, even though the exact mechanism remained unclear. For example, stem cell-based therapy has been considered as an attractive new therapeutic approach for acute lung injury. Multiple paracrine factors produced from stem cells were proposed to play critical and necessary therapeutic roles in the maintenance of endothelial and epithelial integrity, down-regulation of both tissue and circulating inflammation, acceleration of tissue repair process, and/or inhibition of bacterial growth [9]. It is also possible that endogenous lung stem cells or cell progenitors may be activated and play an important role in response to various challenges. Recent study provided the evidence to show that human lungs contain undifferentiated human lung stem cells nested in niches in the distal airways, which have capacities of self-renewing, clonogenic, and multipotent in vitro [10]. Such lung stem cells, as the source of endodermal and mesodermal lineages, are different from known regional pools of progenitor cells. This is a new concept for understanding lung biology and the development of new cures for debilitating lung diseases and holds new implications for regenerative medicine to treat lung disease. Those findings can lead to different translational strategies of cell-based therapy to clinical application to treat lung diseases. It is more challenging to translate the experimental efficacy of external stem cells and the existence of human lung stem cells into the therapies and predictions for lung diseases. However, eventually the first cell-based clinical trial in acute lung injury has just been approved by FDA, suggesting new landmark of stem cell research and may hold promise for patients treatment in the near future.

A large number of constantly advancing biotechnologies need to be translated into diagnoses and therapies for respiratory diseases as new applications to benefit patients. Genetic engineering to create tumor-fighting immune cells for cancer therapies has been considered as one of 5 recent breakthrough innovations in biotechnology. The cancer-targeted specific therapy by genetic engineering of the recipient’s immune system could be carried out by transfer of T-cell receptor genes, taken from lymphocytes of a patient who had an immune response to cancer, to lymphocytes from other patients [11]. The feasibility and clinical responses with adoptive transfer of ex vivo generated immunologic effector cells that are capable of targeting metastatic melanoma. The preliminary study in humans provides evidence of the feasibility and antitumor activity of genetically modified lymphocytes achieved by high-capacity viral vectors, with optimization of the approaches. Such advanced biotechnologies make it possible to create genetically engineered adoptive Th2 cells or cancer-targeted T-cell receptor lymphocytes to treat allergic diseases or lung cancer. We are excited and encouraged by those outstanding findings and questioned if such approaches can be translated from other cancers to lung cancer, preclinical trials to clinical application, or potential long-term risks to safe and durable therapies.

Another topic of translational respiratory medicine is to translate the understanding of molecular mechanisms into personalized medicine and therapies and hospital treatments and manipulations into distant electronic medicine (eMedicine). We create and establish a system of distant eMedicine to closely connect people at risk from respiratory diseases with pulmonologists through the cloudy system. People can reach the pulmonologist and report their symptoms and lung function tests in a real time, without the need to come to the hospital. This is a new aspect of translational respiratory medicine from clinical treatment to social prevention, static outpatient clinic to dynamic and real-time monitoring, and a small group of patients with respiratory diseases to a large population of people with any risk of respiratory disorders or discomfort. Without limits, *Translational Respiratory Medicine* will publish papers in all relevant areas designed to further understand the molecular mechanisms underlying organ or cell dysfunction in human pulmonary diseases, dedicated to the discovery and validation of diagnostic and prognostic disease biomarkers, and the identification and validation of novel drug targets, application of tissue genomics, transcriptomics, proteomics and bioinformatics in drug efficacy and toxicity in clinical research.

Translational Respiratory Medicine will refresh the “old” clinical topics, e.g. mechanical ventilation and lung function, and translate clinical challenges and considerations to molecular biologists to innovate and develop new therapies and supports to patients with lung diseases. One way is to identify and validate gene and protein biomarkers from patients with lung diseases for the correlation with lung function measurements, even better to follow up the alternations by time [6]. Or the advanced biotechnologies and molecular understanding should be translated to the clinical research of mechanical ventilation. For example, mitochondrial biogenesis and content, respiratory chain cytochrome c oxidase, mitochondrial DNA deletions, lipid accumulation, AMP-activated protein kinase and sirtuins, mitochondria-derived oxidative stress, and cellular energy status were investigated in human diaphragms after mechanical ventilation, in order to explore the potential mechanisms of ventilator-induced diaphragmatic dysfunction [12]. We need more molecular evidence to describe dysfunction of genes, proteins, cellular organelles, cells, or interactions between and refresh “old” applications of clinical monitors or therapies.

I am pleased and honored to be co-editor of the journal *Translational Respiratory Medicine* with Dr Michael Matthay, a close friend, supportive colleague, and valuable mentor for my post-doctoral education. *Translational Respiratory Medicine* is an additional but unique opportunity for scientists and clinicians who work on pulmonary diseases to publish their outstanding findings, initiative results, and critical and perceptive opinions in the journal. In initiating the journal, we would like to express our special appreciations to all colleagues for their encouragement, support, comments, suggestions and contributions. With the support of our Associate Editors and Editorial Board Members, we have enough reason to believe that *Translational Respiratory Medicine* will be one of the leading platforms to translate basic sciences associated with respiratory diseases into clinical applications and benefit the healthcare of humans.

## References

[1] [http://www.who.int/healthinfo/global_burden_disease/en/index.html]

[2] Abraham E, Marincola FM, Chen ZN, Wang XD (2012). Clinical and translational medicine: Integrative and practical science. Clin Transl Med.

[3] *Specialized Programs of Research Excellence (SPORE) in Gastrointestinal Cancer, NIH GUIDE, Volume 22*. Volume Number 1. 1993. RFA: CA-93–16)

[4] Minna JD, Gazdar AF (1996). Translational research comes of age. Nat Med.

[5] Wang XD (2011). Role of clinical bioinformatics in the development of network-based Biomarkers. J Clin Bioinforma.

[6] Artigas MS, Loth DL, Wain LV, Gharib SA, Obeidat M (2011). Genome-wide association and large-scale follow up identifies 16 new loci influencing lung function. Nat Genet.

[7] Chen H, Wang Y, Bai CX, Wang XD (2012). Alterations of plasma inflammatory biomarkers in the healthy and chronic obstructive pulmonary disease patients with or without acute exacerbation. J Proteomics.

[8] Chen H, Song Z, Qian M, Bai CX, Wang XD (2012). Selection of disease-specific biomarkers by integrating inflammatory mediators with clinical informatics in AECOPD patients: a preliminary study. J Cell Mol Med.

[9] Lee JW, Fang X, Krasnodembskaya A, Howard JP, Matthay MA (2011). Concise review: Mesenchymal stem cells for acute lung injury: role of paracrine soluble factors. Stem Cells.

[10] Kajstura J, Rota M, Hall SR, Hosoda T, D'Amario D, Sanada F, Zheng H, Ogórek B, Rondon-Clavo C, Ferreira-Martins J, Matsuda A, Arranto C, Goichberg P, Giordano G, Haley KJ, Bardelli S, Rayatzadeh H, Liu X, Quaini F, Liao R, Leri A, Perrella MA, Loscalzo J, Anversa P (2011). Evidence for human lung stem cells. N Engl J Med.

[11] Koya RC, Mok S, Comin-Anduix B, Chodon T, Radu CG, Nishimura MI, Witte ON, Ribas A (2010). Kinetic phases of distribution and tumor targeting by T cell receptor engineered lymphocytes inducing robust antitumor responses. Proc Natl Acad Sci U S A.

[12] Picard M, Jung B, Liang F, Azuelos I, Hussain S, Goldberg P, Godin R, Danialou G, Chaturvedi R, Rygiel K, Matecki S, Jaber S, Des Rosiers C, Karpati G, Ferri L, Burelle Y, Turnbull DM, Taivassalo T, Petrof BJ (2012). Mitochondrial Dysfunction and Lipid Accumulation in the Human Diaphragm during Mechanical Ventilation. Am J Respir Crit Care Med.

